# Women with infertility complying with and resisting polygyny: an explorative qualitative study in urban Gambia

**DOI:** 10.1186/s12978-019-0762-1

**Published:** 2019-07-15

**Authors:** Susan Dierickx, Gily Coene, Bintou Jarju, Chia Longman

**Affiliations:** 1 Centre of Expertise on Gender, Diversity and Intersectionality (RHEA), Vrije Universiteit Brussel, Pleinlaan 2, 1050 Elsene, Belgium; 2 Centre for Research on Culture and Gender, Ghent University, Rozier 44, 9000 Ghent, Belgium; 3 Vaccines and Immunity Theme, Medical Research Council Unit The Gambia at the London School of Hygiene and Tropical Medicine, Fajara, The Gambia

**Keywords:** Childlessness, Coping, Infertility, Polygyny, Polygamy, Harmful cultural practice, Resistance, The Gambia, Women's rights

## Abstract

**Background:**

In many low-and middle-income countries women with infertility are often in polygynous marriages. From a human and women’s rights perspective, the practice of polygyny is commonly understood as harmful. Studies indicate that polygyny aggravates negative life circumstances of women with infertility with respect to their health and social well-being. The purpose of this qualitative study is to explore how women with infertility experience polygyny and to understand their decision-making regarding these marriages.

**Methods:**

An explorative qualitative study was conducted among women with infertility in the urban communities of the West Coast region of The Gambia using in-depth interviews (30). Data analysis involved an emergent and partially inductive thematic framework and was carried out using NVivo 11.

**Results:**

With the exception of some women with infertility who described positive experiences within polygynous marriages, most women emphasised conflicts that exist within polygynous households and reported financial and emotional difficulties. Thematic analysis identified several strategies of women with infertility to cope with and resist polygynous marriages, including overcoming childlessness, addressing conflict, spending time outside the compound, looking for social support, *kanyaleng kafoolu*, living separately and initiating divorce. Moreover, the experiences and decision-making power of women with infertility when it comes to polygynous marriages was found to be closely related to their socio-demographic background.

**Conclusion:**

This work highlights how women with infertility in polygynous marriages are in a precarious situation in urban Gambia. Women utilize a mix of compliance, coping and resistance strategies to navigate the challenges of polygynous marriages in a structurally constraining context.

## Plain English summary

In many low-and middle-income countries women with infertility are often in polygynous marriages (i.e. marriages wherein men have multiple wives). In general, the practice of polygyny is understood to be harmful for women as it would have a negative impact on their health and general well-being. The purpose of this qualitative study is to explore how women with infertility experience polygyny and to understand their decision-making regarding these marriages. In-depth interviews were conducted between October 2017 and May 2018 in urban Gambia. With the exception of some women with infertility who described the positive impact of polygyny on their daily lives, most women discussed the conflicts that exist within polygynous households and the financial and emotional difficulties. We identified several strategies of women with infertility to deal with polygynous marriages, including overcoming childlessness, addressing conflict, spending time outside the compound, looking for social support, *kanyaleng kafoolu* (i.e. groups for women who experience infertility and child death), living separately and initiating divorce. Most women thought it was unlikely that the practice of polygyny would change. Women recommended the implementation of interventions that foster their empowerment and that would improve access to reproductive health care.

## Background

Infertility is a global reproductive health problem, affecting an estimated 48.5 million couples worldwide [1]. Not only is infertility a reproductive health problem, for many people around the world infertility also constitutes an important aspect of their lives [2–8]. In several Sub-Saharan (SSA) countries (including The Gambia) infertility problems are often attributed to women regardless of medical diagnosis [6, 7, 9–19]. As in other SSA countries, Gambian women with infertility are confronted with general emotional distress, financial difficulties, stigmatization and isolation at the community level [6, 9, 12, 15, 20, 21]. Studies have shown how the impact of infertility on women’s marriages is diverse. Women with infertility often face more verbal and physical violence, divorce, extramarital affairs and polygyny (i.e. men marrying multiple wives) [9, 22–24]. The experience of polygyny has not been discussed at length but is often mentioned as one of the hardships women with infertility endure [9, 11, 12, 21–25].

Scholars reporting on the experiences of women with infertility in polygynous marriages can broadly be categorised into two groups, based on opposing conceptions of agency and victimisation. Those who hold the victimisation perspective generally perceive women with infertility in polygynous marriages as sufferers [26, 27]. This perspective follows the view of human and women’s rights organizations describing polygyny as inherently harmful to women, a ‘backward’ tradition or religious practice and a cause of underdevelopment [27–36]. Scholars and activists point out that polygyny can have considerable detrimental impact on women’s well-being. Several studies report how polygyny is related with a lower uptake of contraceptives and an increased chance for sexually transmitted infections (STIs), including HIV/AIDS [6, 22, 27, 28, 34, 37–40]. It has also been suggested that polygyny may contribute to fertility problems given the irregularity of sexual contact and a higher susceptibility to STIs (i.e. one of the main causes of infertility in SSA) [41]. Polygyny also has an indirect impact on women’s health, as a study conducted in rural Gambia found there were fewer resources to access health care in polygynous marriages [42]. Women in polygynous relationships tend to have less power and are therefore more likely to suffer from physical and psychological abuse within their marriages [27, 43] and experience higher levels of anxiety and depression [23, 38].

In contrast, several anthropologist and feminist scholars who apply an ‘agency’ perspective argue that women in low-and middle-income countries are too often perceived as passive victims of culture and traditions [44–47]. Several authors have argued that the practice of polygyny itself should not be blamed, but rather men’s misappropriation of the privileges of the custom while rejecting the responsibilities [48, 49]. Any analysis of polygyny should consider the local specificity of gender and kinship arrangements, and attend to how the complex inner dynamics of polygynous households may play out for the women (and men) concerned [26, 36, 48–50]. Although from a Western perspective, polygyny might be indicative of relational problems and the suffering of women with infertility, this can be in conflict with the perceptions of women with infertility themselves [16]. For example, Nwoye [49] states that polygyny can be a means to deal with infertility among the Igbo and the Yoruba in Nigeria, the Luo people of Kenya and the Acholi people of Uganda. In these contexts, the co-wife can be seen as a comrade with the objective of bringing children into the household. In The Gambia, anecdotal evidence suggests that the institution of polygamy has rarely been questioned by women with infertility [51]. This last study also shortly describes how many women with infertility have an ambivalent attitude towards their co-wives: sometimes co-wives are accused of witchcraft but in other situations, foster children are given to the wife with infertility suggesting a more positive relationship.

This research is part of a larger study on the experiences of women with infertility in The Gambia (West Coast region) and Senegal (Casamance). The current study starts from a dynamic view on culture and society that allows to take account of different perspectives and experiences on polygyny [45, 52–56]. Based on qualitative research, this paper aims to explore the experiences of women with infertility regarding polygynous marriages in the urban communities of the West Coast region of The Gambia. A qualitative approach allows for an in-depth understanding of how people construct the world around them, thereby providing insight into what they are doing and what is happening to them in meaningful ways [57]. The research seeks to address the following questions:

(i) how do women with infertility experience polygynous marriages?
(ii) What is the decision-making power of women with infertility when it comes to polygynous marriages? More specifically,
  - to understand the involvement of women with infertility in the decision-making to engage in polygyny;
  - to identify which strategies women with infertility use to cope with polygyny;
  - to identify which strategies women with infertility use to resist polygyny.

## Methodology

### Study setting

The study is based on a qualitative enquiry carried out intermittently between October 2017 and May 2018 (from 26/09/2017 until 23/10/2017; from 8/11/2017 until 6/12/2017; from 18/01/2018 until 12/2/2018; from 13/3/3/2018 until 10/4/2018) in the urban communities of the West Coast region of The Gambia. In the urban area, the Mandinka and the Wolof are the largest ethnic groups but the population is very diverse given the migration from the rural to the urban regions [58]. In the country, Islam is the pre-dominant religion (90%), but there are also Christians (8%) and a minority (2%) exclusively follows traditional religions. A virilocal residence pattern is common whereby the newly-wed moves into her new marital family home where she lives with the extended family members of her husband [12, 59].

Currently polygyny in The Gambia is often associated with Islam, but historical analysis shows that polygyny predates Islam in West Africa [60]. The Gambia has a tripartite legal system of legislative, customary and Islamic or Sharia law, recognizing four types of marriages: Christian, Muslim, customary and civil marriages [61]. Customary and Islamic marriages allow polygyny. Most Gambian marriages are customary. Polygynous marriages are described as a normative marital institution in the country, with the most recent Demographic Health Survey showing high levels of polygynous marriages (39%) [42, 58, 62]. This data further indicates that older women are much more likely to be in polygynous unions than younger women. Women who are better educated and have a higher socio-economic status are less likely to be in polygynous marriages.

### Sampling, recruitment and sample characteristics

When analyzing infertility, it is important to acknowledge that standard epidemiological and demographical conceptualizations of infertility are not always meaningful for people living in low- and middle-income countries [12, 63–65]. Conceptualizations about what is natural, normal or expected in terms of fertility varies in time and over situation. Therefore, this study defined women as infertile when they considered themselves as such, regardless of the duration of their fertility problems and their number of living children. This study included women regardless of their current marital status since previous research has pointed out that Gambian women with infertility who are currently in a monogamous marriage might experience mental stress about the possibility that their husband will engage in polygyny or could have been previously in a polygynous marriage, making their perspectives and experiences relevant for this study [12]. Women with infertility were (i) identified at community level; (ii) with the assistance of local organizations; and through (iii) snowball sampling, whereby respondents identified other potential respondents.

Of the 30 women with infertility who were interviewed, 19 were currently in polygynous marriages (Table 1). The socio-demographic characteristics had an influence on the type of marriages women are engaged in. Some of the interviewed women were in their second or third marriage because (i) couples divorce and remarry after their first marriage remained childless or (ii) many of the older respondents were married as minors (from the age of 14 onwards) or early adults, sometimes with much older men who passed away or from whom they divorced.

**Table 1:** Overview of socio-demographic characteristics women with infertility

Respondent	Data source	Age^a^	Schooling level	Profession	Ethnicity	Current marital status	Times married
1	Interview	Adult	Master abroad	Civil servant	Wolof	Monogamous marriage	1
2	Interview	Adult	Master abroad	Civil servant	Wolof	Monogamous marriage	1
3	Interview	Adult	No	Petty trading	Fula	Polygynous marriage	1
4	Interview	Adult	No	Vendor market	Mandinka	Polygynous marriage	3
5	Interview	Elder	No	Housewife	Mandinka	Widow (monogamous marriage)	1
6	Interview	Elder	No	Vendor market	Mandinka	Polygynous marriage	2
7	Interview	Elder	No	Vendor market	Masuwanka	Polygynous marriage	1
8	Interview	Elder	No	Vendor market	Mandinka	Polygynous marriage	2
9	Interview	Elder	No	Farmer	Fula	Polygynous marriage	2
10	Interview	Adult	No	Vendor market	Wolof	Polygynous marriage	2
11	Interview	Elder	No	Housewife	Wolof	Monogamous marriage	1
12	Interview	Elder	No	Vendor market	Mandinka	Polygynous marriage	2
13	Interview	Adult	No	Housewife	Mandinka	Polygynous marriage	1
14	Interview	Elder	No	Vendor market	Mandinka	Polygynous marriage	2
15	Interview	Elder	No	Petty trading	Serer	Polygynous marriage	1
16	Interview	Adult	No	Petty trading	Mandinka	Polygynous marriage	1
17	Interview	Adult	No	Housewife	Mandinka	Polygynous marriage	1
18	Interview	Adult	No	Housewife	Mandinka	Polygynous marriage	1
19	Interview	Adult	High school	Housewife	Mandinka	Monogamous marriage	1
20	Interview	Adult	No	Vendor market	Mandinka	Polygynous marriage	3
21	Interview	Adult	No	Vendor market	Mandinka	Polygynous marriage	3
22	Interview	Elder	No	Housewife	Mandinka	Widow (Polygynous marriage)	1
23	Interview	Elder	No	Vendor market	Mandinka	Polygynous marriage	2
24	Interview	Elder	No	Housewife	Mandinka	Widow (monogamous marriage)	1
25	Interview	Adult	No	Cleaner	Mandinka	Monogamous marriage	1
26	Interview	Elder	No	Vendor market	Mandinka	Polygynous marriage	2
27	Interview	Adult	Primary school	Petty trading	Mandinka	Widow (polygynous marriage)	1
28	Interview	Elder	No	Housewife	Mandinka	Polygynous marriage	1
29	Interview	Adult	No	Farmer	Mandinka	Monogamous marriage	2
30	Interview	Elder	No	Private business	Mandinka	Monogamous marriage	1

^a^Adult: 18–49 years old/Elder: + 50 years old

### Interviews

Semi-structured in-depth interviews were conducted with 30 women with infertility on the topic of marriage. When appropriate, respondents were interviewed multiple times in order to gain a more in-depth understanding. Most interviews were recorded. However, if the interviewee preferred not to be recorded, extensive notes were taken during the interview or directly afterwards. Interviews lasted on average 45 min and were conducted at private places where respondents felt at ease such as the privacy of their residences, the houses of trusted friends, or in a room at a local organization working with women with infertility. The researcher and translator made sure that nobody could overhear the conversation. In-depth interviews were conducted by the first author and took place in English if the respondents were fluent in this language. This was the case with most financially independent women with infertility who were highly educated. In other cases, data collection was carried out with the assistance of a local female translator who translated the questions of the first author into the local language. This was done if respondents were not speaking English or if they preferred to discuss this sensitive topic in their local language. Prior to data collection, the translator assisting the first author received training in research facilitation, interview skills and confidentiality protection. The translator was supplied with the question guides in order to discuss terminology, phrasing and translation of potential questions. During data collection, attention was paid to non-verbal communication such as body language, gestures and silences.

### Data analysis

Data analysis was an emergent and inductive coding process in order to grasp the context in which human experience occurs [66]. The analysis done by the first author consisted of repeatedly reading each of these transcripts, identifying statements and quotes, and then labelling and connecting these to particular themes (e.g. divorce, inheritance). These were then analysed interpretatively into higher-order themes (e.g. resistance strategies, experiences of financial challenges) according to their conceptual coherence resulting in a coding tree in NVivo 11 Analysis Software (QSR International Pty Ltd. Cardigan UK). To limit potential bias, respondents were encouraged to participate in the analysis process by inviting them to explain preliminary findings and contradictions during interviews [56]. Discussion of preliminary findings showed that the results found resonation with how participants experienced their reality. The first author received feedback from senior authors based on analytic discussions, which informed the final interpretations as presented here.

### Reflection about positionality

Reflexivity about the influence of the positionality of the researcher and translator on data collection and research ethics was important during all stages of the interview process. Prior to the current research project, the first author resided in total 14 months during 3 years (2012–2015) in The Gambia doing research on health and pregnancy together with the translator. From previous stays the first author had learned that worries related to reproduction were part of the everyday lives of Gambian people, as they are at the core of many people’s lives. She also resided with a Gambian family allowing her to closely observe and participate in everyday activities. These experiences made her acquainted with culturally appropriate expressions and behaviour, making people more at ease to share their thoughts and experiences with her. The first author and the translator are women which might have led to more openness about the challenges of polygynous marriages [38]. At the same time the position of the first author as a foreign researcher made her a confidential conversation partner for those wanting to share their stories. The translator was a married Gambian woman who had a child, this was important as previous research experience learned that women were less likely to talk about their experience to unmarried women. The translator was living in the urban area but not in the same communities as the participants. At the beginning of each in-depth interview she identified herself as a translator and promised confidentiality to respondents.

### Ethical approval

The study was approved by The Gambia Government/MRC joint Ethics Committee (SCC 1562) and by the Ethical Commission of Vrije Universiteit Brussel (ECHW_096) (Belgium). The interviewers followed the Code of Ethics of the American Anthropological Association (AAA). This implied that all interviewees were informed about the purpose of the study, the topic and type of questions as well as their right to decline participation or to interrupt the conversation at any time. Anonymity was guaranteed, and confidentiality of interviewees assured. If people consented to be interviewed, respondents were asked to provide written consent and during each in-depth interview they had the opportunity to ask questions about the research.

## Results

The first section starts with an overview of the positive and negative experiences of women with infertility in polygynous marriages. Next, the decision-making power of women in polygynous marriages is discussed looking into their influence in the set-up of polygynous marriage, and with respect to their coping and resistance strategies.

### The experiences of women with infertility in polygynous marriages

#### Cooperation and practical support

Societal norms prescribe that co-wives should be treated equally by both the first wife (also often called the senior wife) and the husband. This translates itself into strict arrangements about sleeping patterns and cooking habits. There were many women with infertility who said they had a good relationship with their co-wives who respected and supported them with their daily household chores enabling them to engage in activities outside the compound. They also expressed that they had a good relationship with their husband. These women regularly referred to their faith and situated the practice of polygyny in a religious framework: *‘The prophet has four wives and we are following in his footsteps’* (Respondent 28).

#### Perceived influence on fertility and sharing of children

Women with infertility explained that *‘the womb is jealous’*. This common expression means that when a co-wife or someone close to the woman with infertility becomes pregnant it increases the likelihood to become pregnant. In the study, there was one respondent who said she became pregnant when her husband married a second wife and therefore she experienced the second wife as a blessing. Two interviewed women with infertility said that they were given as a baby by their mother to their mother’s co-wife with infertility. However, nobody in this study fostered children of their own co-wife.

#### Conflicts within the household

*‘The worst is if you don’t have a child and he marries another wife who starts having children. It is painful. The wives would start to say many things. The one without children will say that the husbands’ concentration is with the one with kids and vice versa.’* (Respondent 6)

In general, women with infertility agreed that within polygynous households there are many tensions and disputes between the different parties involved: the husband, the co-wives and the children. While women commonly mentioned verbal conflicts, few admitted that they experienced physical violence from the co-wife or husband. The most common complaint is that women with infertility receive less love and attention from the husband. When the woman with infertility was the first wife of the husband, it might be difficult for her to sustain the culturally expected position as the manager of the household. This is explained by the already weak position of the woman in the household resulting from fertility problems. There is also the chance that the husband might divorce his wife with infertility after the co-wife has children.

One way in which tensions are expressed are in accusations about the use of witchcraft and evil spells by *marabouts* (i.e. indigenous healer who can also cause misfortune). An interviewee narrated how she suspects that her co-wife went to the *marabout* to poison their husband, while another woman thought her complications during labour were caused by an evil spell by a *marabout* at the request of her co-wife. In other instances, women with infertility were accused by their co-wife of being witches.

#### Financial difficulties

Many interviewed women disapproved of polygynous marriages because of financial problems. Cultural norms prescribed that men should provide *fish money* (i.e. money for the daily livelihood) for each of his wives. Yet several women with infertility complained they did not receive any fish money. Some divorced or widowed women with infertility often didn’t even harbour the expectation to be financially supported by their new husband.

*‘My new husband is also having another family [ … ] When my husband passed away, he told me that he wanted to marry me and I accepted, but he also made it clear that he cannot provide all what I need [ … ] It is better than not having a husband. Whenever he has money, he provides us with food and shelter.’* (Respondent 6)

When the husband did support each wife, the same amount of financial resources was shared among more people. This implies that the husband also had fewer financial means to support his wife with infertility. When women were financially independent or the husband had a good job, this did not have to be problematic. However, many of the interviewed women in polygynous marriages had a lower socio-economic status and the financial implications of polygyny were experienced as a hardship. Lastly, in polygynous marriages women with infertility were in a vulnerable position when their husband died. The local interpretation of Sharia law was often followed closely when it came to inheritance. This implied that sons received at least double the share of daughters and wives. Upon their husband’s death, widows without children could be expelled from the compound by their in-laws or co-wives.

#### Emotional challenges

Respondents agreed that becoming a co-wife when you are not able to have children could lead to many psychological difficulties. In addition to the aforementioned conflicts within the household and financial burdens, women with infertility experienced it as very painful to see the children of their co-wife growing up. They also expressed feelings of jealousy and sadness. Women with infertility talked about a negative self-image and stress in polygynous marriages: ‘*When I go to the compound I get sad and angry because of the attitude of my co-wife’* (Respondent 10).

### Decision-making of women with infertility

#### The role of women in deciding for a polygynous marriage

Making an agreement about polygyny before getting married remains difficult for women even when they are highly educated and have a secure job. It was explained that men often referred to their religious right and duty to have multiple wives. Women with infertility described how in the broader society it is perceived to be normal for men to get a second wife when the first one is not able to have children or if there is no son who can continue the family lineage. Several women associated their husband’s decision to engage in a polygynous marriage with their fertility problems.

In this study, three women with infertility encouraged their husband to take another wife. The first respondent said she wanted to have a co-wife so she would be able to share the responsibilities of the household and have more freedom to travel. The second woman wanted her husband to remarry since she was beaten severely due to her infertility and hoped this would decrease the pressure on her to become pregnant, but her husband never remarried. The last woman expressed how her husband supported her during her fertility problems and said that she hoped that having a co-wife would make her womb jealous.

Several women with infertility in polygynous marriages were not informed about their husband’s decision to marry a second, third or fourth wife until the day of the marriage itself, which was an upsetting experience: *‘My husband knows that more than me, sometimes he would marry them without letting me know’* (Respondent 15). In most situations, the decision to engage in polygyny was made by the husband, sometimes under pressure of his family who also find it very important that their brother or son has children.

#### Coping strategies of women with infertility

The data indicated there were several strategies women with infertility employed to cope with the challenges in polygynous marriages.

##### Improving social position by having children

An important strategy for women with infertility in coping with the challenges that polygynous marriages bring, is to improve their status by overcoming their childlessness. Many women are looking for health care, both indigenous as biomedical. Some women discussed how their husbands provided financial and emotional support, but mentioned that they would rarely go for diagnosis. In most instances, when husbands are in polygynous marriages the financial support decreases or stops because of the many competing demands from other household members. In addition, children can be given by kin and close friends to women with infertility outside the legal framework. It was recurrently mentioned that fosterage is a widely accepted practice in The Gambia that happened because of multiple reasons e.g. to help people with infertility, to strengthen the relations between birth and foster parents, to solve practical problems in child care or to provide a home to children whose parents died or divorced. Fosterage may provide women with children who can provide domestic help, company and affection. However, it is often experienced as a second-best option as there is no guarantee that the child remains loyal and will support the foster parent in old age. Though it is considered to be cruel to take children away from women with infertility, a few interviewed women experienced this which they still felt difficult to handle:

*Of all of those I fostered, two of them when they were about to complete high school, they transferred to their mothers’ house. Now the children here with me are the children of my sisters and my co-wife ( … ) I really feel sad about that. If they were my own children they would not have gone.*

##### Addressing conflict

Some women with infertility discussed how they tried to connect with the co-wife; establishing a good relationship despite the challenging experiences. Children of the co-wife could be brokers in these relationships. Some respondents mentioned they would talk with their husband to discuss the challenges they were facing.

##### Looking for social support

Women with infertility indicated during interviews how they looked for social support from friends, family and neighbours. These people helped in several ways; some women said they talked about the challenges they experienced with their co-wives, other times these people provided a second home where they could stay for longer periods escaping the challenges at home. In other situations, these people mingled in the household of the women with infertility, for example by protecting the woman with infertility when a co-wife became physically or verbally aggressive.

##### Spending time outside the compound

Several women with infertility living in a polygynous marriage escape the tensions by spending time outside the compound. Interviewed women with infertility having their own business or professional job could escape tensions at home during their working hours. Women without a professional job often spent their time working on their farms or at the market.

##### *Kanyaleng kafoolu*

*Kanyaleng kafoolu* are groups consisting of women who experience infertility and child death. Our research showed how these organizations provide social, emotional and sometimes financial support for women who have been unable to achieve the local reproductive norm of a large family. Mostly women from a lower socio-economic background were *kanyalengs.* Being a *kanyaleng* allowed them to escape the stress they experienced in their daily life. Many *kanyalengs* are very active outside their compound as they hold their own initiation rituals. They also prepare food, dance and sing during public events in the community such as naming and wedding ceremonies. Women with infertility explained during interviews that during the last 10 years, some groups are being paid to work as ‘traditional communicators’ for organizations with development and health agendas because of their skills in song and dance.

##### Living separately

In a few instances, co-wives live in different compounds to decrease tensions. This could happen when the husband has enough financial means, or when he has two or more houses, for example a house in the place where he works and another in the village he originates from. In those instances, it occurred that co-wives didn’t even know each other. One story was shared about a woman with infertility who was granted her house from her brother. Most of the time she lives there alone, and when there is a fight with her husband she can send him away from her house giving her some independence in the relationship. One interviewed woman with infertility described her second marriage, in which she has the position of fourth wife, as follows: *‘There is not a lot of love, but the situation favoured me because I am living in my family compound [i.e. the compound of her father].’* (Respondent 9).

##### Resisting polygynous marriages

*In private life*. Women who were described as ‘strong’ were less inclined to accept polygyny which appeared to have an influence on their husband’s decision to engage polygyny. The concept of ‘strong women’ is used to refer to well-educated and financially independent women, but also to women who have a very outspoken opinion. Women with infertility could divorce their husband when he engaged in polygynous marriages, but this is rare because (i) there is a strong social pressure to stay married and accept polygynous marriages and (ii) divorced women are likely to become a co-wife in their next marriage especially when they have fertility problems. Women with a higher socio-economic status and more choices regarding their welfare and marriage were more likely to divorce their husband.

During interviews women who were critical about polygyny commonly referred to their religion.

*‘I don’t think it’s good to marry a new wife when the first wife is having infertility problems. To have a child is the will of God.’* (Respondent 15)

Furthermore, some educated women argued that their husbands’ polygynous marriages did not follow Islamic regulations. They discussed how the Prophet said that all wives should be taken care of and treated equally by their husbands, which did not happen in practice. They would say that in many polygynous marriages the *‘heart cannot be equal’* and men did not financially support their wives.

*In public space*. When asked about any societal changes when it comes to polygyny, a few women said that the prevalence of the practice and the number of co-wives is decreasing which they associated with the empowerment of women through education. Yet most respondents mentioned that nothing has changed when it comes to polygyny and that it was unlikely to change in the future because people saw it as part of their religion and culture. Religious discourses around polygyny emphasize the practice as a divine right for men making it a normative practice in the country and difficult to contest in public. Some women remarked that any intervention on the topic would be seen as an intervention from ‘outsiders’ and unlikely to be effective. This contrasts with some women’s involvement and strong opinions about other practices considered to be harmful by the international community such as child marriages. Several women said that interventions should not focus on polygyny, but on the implementation of income generating activities and the institutionalization of centres where women can learn new skills which would empower them. Most women also explained that infertility was the major concern in their lives and urged for better access to reproductive health care to prevent and cure infertility.

## Discussion

The objective of this study was (i) to explore how Gambian women with infertility living in the urban areas of the West Coast region experience polygyny, and (ii) to ascertain their decision-making regarding polygynous marriages.

The Gambian context is characterized by a strong pro-natal norm and high levels of gender inequality. Polygyny is understood as a normative practice, especially when women are infertile [12, 67]. The perspectives and experiences of women with infertility are lost and misunderstood if they would not be situated in this specific context. In urban Gambia few women with infertility had positive experiences with polygynous marriages and it was agreed that, in general, tensions, disputes and financial worries are common within polygynous households which led to emotional challenges. Women with infertility also have limited choices, for example many of them were not involved in their husband’s decision to engage in polygynous marriages and had to accept the marriage as a matter of fact. However, the data showed that binary victim/agent conceptualizations of women with infertility in the literature are inadequate. The portrayal of women with infertility in polygynous marriages as passive victims would oversimplify their situation and even contribute to their stigmatisation. Rather, it is more productive to consider women with infertility as active decision-makers, using multiple coping and resistance strategies to handle various situations.

There are women with infertility encouraging their husband to engage in polygyny because of pragmatic reasons. Other women with infertility actively discouraged their husband to engage in polygyny and remained in monogamous relationships. The ability of women with infertility in polygynous marriages to address problems arising from these unions is illustrated in the identified coping and resistance strategies, i.e. overcoming childlessness, addressing conflict, spending time outside the compound, looking for social support, the institution of the *kanyaleng kafoolu*, living separately and divorce. Based on their experiences, women were struggling to make their marriages work or to improve their general well-being. Thereby, it should be noted that women with infertility with a high socio-economic status, who are formally schooled and have a strong social network were more likely to employ certain coping and resisting strategies. This is not surprising as they have a stronger position within their marriage and broader social networks [12]. However, independent of socio-economic status, those women who are described as *strong*, referring to their very outspoken opinion, also confronted their husbands with the potential problems of polygyny.

Interestingly, many women with infertility referred to their religion to explain and support the practice of polygyny, but some also referred to religion to contest it. Women with infertility who contested the practice of polygyny based on religious arguments explained that men commonly didn’t follow Islamic guidelines regarding the equal treatment of wives and weren’t able to take care of their wives. Women also found support in their religion when faced with infertility; they perceived their husband’s decision to take a co-wife as an unjust punishment for a situation that was God’s will. These findings resonate with the view of anthropologists and feminist scholars stressing that culture and religion can be both a source of oppression and support for women [44, 45, 54, 55]. Individuals are able to contest, change and conform to aspects of their culture and religion, resulting in diverse and sometimes contradictory experiences and discourses [53].

Although, in general, polygynous marriages had a negative influence on the social, financial and emotional well-being of many women with infertility, the practice is not a topic of public debate. This can be explained by the centrality of religion in Gambian people’s lives and the normativity of polygyny [67]. As Gruenbaum [68] argues on the topic of harmful cultural practices, people should be consulted on what they consider to be harmful and attention should be brought to the broader conditions that serve as obstacles for people’s well-being. In urban Gambia, most of the interviewed women with infertility thought it was unlikely that the practice would change and found infertility the major challenge in their life. They wanted to have more opportunities to empower themselves and better access to reproductive health care to prevent and cure infertility.

A strength of this study is that, being a qualitative study, it delves into real life experiences showing the complexities of polygynous marriages from the perspective of women with infertility. A limitation of the present study is the exclusion of perspectives and experiences of men on polygynous marriages and how this relates to notions of masculinity. In particular, in the Gambian context were infertility is a priori constructed to be a woman’s problem, additional research should be conducted on the experiences of men with infertility and the pressure family members might put on them to engage in polygynous marriages [12]. Furthermore, it should be noted that this study was not intended to compare the situation of women with infertility in monogamous versus polygynous relationships. In The Gambia there is a strong pro-natal norm and female identity is often equated with motherhood and experiences of emotional neglect, mental health problems, physical abuse, (fear of) divorce and extramarital relationships occur both within monogamous and polygynous marriages [12]. All women with infertility, therefore, would benefit not only from better access to treatment, but also from counselling. More research should be carried out on how to develop counselling programs in a culturally-sensitive way.

## Conclusion

This study highlights how polygyny is a complex topic and normative practice in The Gambia. The findings suggest that a balanced discussion of women’s experiences moves beyond the paradigm of victimhood [54, 69, 70]. Portraying women with infertility in polygynous marriages as merely victims would oversimplify their situation and contribute to their stigmatisation. Given the structural constraining context of gender inequality and pro-natal norms, women with infertility are neither completely free agents nor passive victims. In general, the study indicated that polygyny did have a negative influence on women’s financial, social and emotional wellbeing. The complexity of challenges that women with infertility in polygynous marriages experience, as well as their decision-making abilities, requires more attention in qualitative and quantitative research. Several coping and resistance strategies (i.e. overcoming childlessness, addressing conflict, spending time outside the compound, looking for social support, the institution of the *kanyaleng kafoolu*, living separately and divorce) are identified which were used by women to navigate the challenges of polygynous marriages, but most interviewed women thought the practice was unlikely to change. Instead of urging for projects and interventions targeting polygyny, they were interested in the implementation of projects that would empower them and that improved access to reproductive health care. Politics and programs focussing on infertility should start from the realities, aspirations and needs identified by women with infertility.

## Data Availability

This study analyses qualitative data. The datasets generated and/or analysed during the current study are not publicly available because the participants did not consent to have their full transcripts made publicly available. The NVivo database with excerpts of the transcripts relevant to the study is available from the corresponding author on request.

## Abbreviations

SSA: Sub-Saharan Africa
STI: Sexually Transmitted Infection
